# Incidental Finding of a Homozygous p.M348K Asymptomatic Italian Patient Confirms the Many Faces of Cystic Fibrosis

**DOI:** 10.1155/2015/289627

**Published:** 2015-04-01

**Authors:** Rossana Molinario, Sara Palumbo, Paola Concolino, Sandro Rocchetti, Roberta Rizza, Giovanni Luca Scaglione, Angelo Minucci, Ettore Capoluongo

**Affiliations:** Laboratory of Clinical Molecular and Personalized Diagnostics, Department of Laboratory Medicine, University Hospital “A. Gemelli”, 8 Largo A. Gemelli, 00168 Rome, Italy

## Abstract

Cystic fibrosis (CF; OMIM number 219700) is an autosomal recessive disease caused by mutations in the *CFTR* (cystic fibrosis transmembrane conductance regulator) gene, which results in abnormal viscous mucoid secretions in multiple organs and whose main clinical features are pancreatic insufficiency, chronic endobronchial infection, and male infertility. We report the case of a 47-year-old apparently normal male resulting in homozygosity for the mutation p.M348K from nonconsanguineous parents. The proband was screened using a standard panel of 70 different tested on NanoChip 400 platform. The massive parallel pyrosequencing on 454 JS machine allowed the second level analysis. The patient was firstly screened with two different platforms available in our laboratory, obtaining an ambiguous signal for the p.R347P mutation. For this reason we decided to clarify the discordant result of *CFTR* status by Next Generation Sequencing (NGS) using 454 Junior instrument. The patient is resulted no carrier of the p.R347P mutation, but NGS highlighted a homozygous substitution from T>A at position 1043 in the coding region, causing an amino acid substitution from methionine to lysine (p.M348K). Casual finding of p.M348K homozygote mutation in an individual, without any feature of classical or nonclassical CF form, allowed us to confirm that p.M348K is a benign rare polymorphism.

## 1. Introduction

Cystic fibrosis (CF; OMIM number 219700) is an autosomal recessive disease caused by mutations in the* CFTR* (cystic fibrosis transmembrane conductance regulator) gene, which results in abnormal viscous mucoid secretions in multiple organs and whose main clinical features are pancreatic insufficiency, chronic endobronchial infection, and male infertility [1]. In recent years it has been acknowledged that a wide clinical spectrum of diseases is associated with* CFTR* mutations. About 10% of the patients present with a mild form of CF, mild respiratory symptoms, pancreatic sufficiency associated to normal, or borderline sweat test results [2]. Since the clinical features of CF are highly variable, the diagnosis of the CFTR-related disorders (CFTR-RD) may be very complex.

Until now more than 1900 different mutations have been reported, with distributions varying among populations (http://www.genet.sickkids.on.ca/app). In Italy, the CF incidence is approximately 1 : 2,700 individuals indicating a rate of carrier individuals of about 1 : 25 [3].

We report the case of a 47-year-old Italian male who was addressed to our department for being submitted to* CFTR* mutation screening, preliminary to an in vitro fertilization procedure, since his partner presented a personal history of reduced ovarian reserve.

The patient was screened using two different platforms available in our laboratory, obtaining an equivocal result for the p.R347P mutation. For this reason we decided to verify the CFTR status by Next Generation Sequencing (NGS). This innovative molecular approach revealed the homozygosity for the nucleotide substitution (c.1043T>A), causing the amino acid change of methionine to lysine at position 348 (p.Met348Lys). This sequence variation is not included in the current* CFTR* mutation screening panel recommended by the American College of Medical Genetics and American College of Obstetricians and Gynecologists. During the subsequent posttest counseling the patient did not report any specific clinical sign associated with CF.

In this study, NGS allowed us to identify for the first time an Italian patient with homozygous amino acid substitution, p.M348K. In addition, in this context, we underline that the use of the NGS for the molecular analysis of the* CFTR* gene can help to expand the spectrum of the* CFTR* mutations facilitating the identification of known, rare, or novel mutations causing the disease, as well as genomic variants that require functional characterization. NGS 454 Junior allowed an accurate and cost-effective approach for the CF genetic testing, suitable for routine clinical practice and ready to be a valid alternative to Sanger sequencing.

## 2. Case Report

A 47-year-old male was referred to our hospital for a checkup before assisted conception. His clinical history was completely negative for (a) consanguinity; (b) infertility, since he is father of a daughter from a previous marriage; (c) pancreatic or acute pulmonary infection; (d) congenital bilateral aplasia of the vas deferens (CBAVD), mild pulmonary expression with bronchiectasis, idiopathic chronic pancreatitis, steatorrhea, hyperbilirubinemia, sinusitis, allergic bronchopulmonary, and asthma. Besides, semen investigation is completely normal. This individual refused to undergo sweat-test since he is often involved in training programs that do not allow performing this type of biochemical evaluation.

Genomic DNA was isolated from peripheral blood by a commercially available CE-IVD kit (High Pure PCR Template Preparation Kits, Roche Diagnostics, USA, http://www.roche.com/index.htm) and the DNA concentration and purity were spectrophotometrically measured (NanoPhotometer, Implen, München, Germany, http://www.implen.de/). Furthermore, the DNA integrity was verified by 0.8% agarose electrophoresis. Initially, the patient was screened using a standard panel of 59 different CF mutations, by reverse dot blots INNO-LiPA* CFTR* 19,* CFTR* 17+ IVS 8 polyT Update, and* CFTR* Italian Regional (Innogenetics, Ghent, Belgium). Dubious INNO-LiPA* CFTR* result was tested with CF70 kit (Nanogen, CA, USA) on NanoChip 400 machine.

NanoChip technology is basically a forward allele-specific oligonucleotide (ASO) based assay which is placed in an electronically controlled microarray format. Each NanoChip consists of 400 spots attached to platinum wire connections. DNA negatively charged is electronically guided to a test site where biotinylated samples bind streptavidin in the site. After denaturation, the CF 70 Data Analysis Spreadsheet indicates the genotype through the Green-to-Red signal ratio for each mutation fluorescent probes (green and red) and signal is detected after stringent washing procedures [4]. For the presence of two discordant results, full coding sequence and exon/intron junctions of the* CFTR* gene were performed by NGS. In particular, we used* CFTR* MASTR v2 assay (Multiplicom, Molecular Diagnostics), following the manufacturer's instructions. Amplicons were purified separately using Agencourt Ampure XP kit (Beckman Coulter) and subsequently quantified with Light Cycler 480 (Roche Diagnostics) by means of the Quant-iT PicoGreen dsDNA Assay kit (Invitrogen). The equimolar pool was amplified to generate an amplicon library using the GS Junior Titanium emPCR Kit (Roche Diagnostics) following the manufacturer's instructions. Four hundred fifty-four sequencing were subsequently performed on a 454 GS Junior v 2.7 system (Roche) using the GS Junior Titanium Sequencing kit (Roche Diagnostics). We employed a new in-house bioinformatics tool, named “*Amplicon Suite*,” able to automatically analyze each single GS Junior Sequencing run. The analysis included different steps: (a) quality control check of sequencing (“coverage” for each* CFTR* amplicon), (b) identification of variants (pathogenic or not), and (c) evaluation of functional effect of variants of uncertain significance (VUS) by processing them with several prediction tools (such as SIFT and PolyPhen). Several filters were applied to discard sequencing errors and to discriminate between polymorphisms and mutations. To confirm the p.M348K mutation, identified by NGS, Sanger sequencing of the* CFTR* exon 8 was carried out. Therefore, DNA sample was amplified using specific primers* CFTR* E8F: 5′-CTCAGGGTTCTTTGTGGTGT-3′ and CFTR E8R: 5′-AATGCCACTCTCATCCATCA-3′. Cycle sequencing reaction was carried out using same primers and Big Dye Termination Kit v. 3.1 (Applied Biosystems). Sequencing PCR products were analyzed using ABI3500 Genetic Analyzer (Applied Biosystems) and aligned to the reference sequences NG_ 016465.1 of the* CFTR* gene.

Preliminary analysis was collected by the Sequencing Software and performed by SeqScape Software v. 3 (Applied Biosystems).

*CFTR* screening by Inno-LiPA kit showed the presence of a weak wild-type band for p.R347P mutation. Since this result was also confirmed on a reextracted DNA sample, the patient was genotyped with an extensive 70 mutation panel through NanoChip technology. In this case NanoChip technology showed a green signal criteria for all* CFTR* markers with a G : R scaled value > 5. This finding highlighted the wild-type genotype for p.R347P mutation. Although this result could be reassuring, the discrepancy between the two technologies suggested a complete screening of* CFTR* gene to detect the possible alteration or mutation due to aberrant reaction found by Inno-LiPA assay.

**Figure 1:**
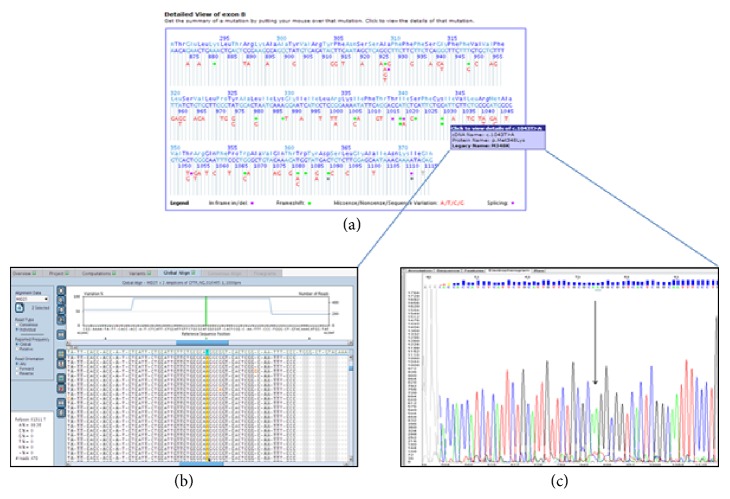
(a) Sequence reference by Cystic Fibrosis Mutation Database (http://www.genet.sickkids.on.ca/app). (b) Amplicon Variant Analyzer v 2.7 (AVA) aligns the sequencing reads to the reference sequences NG_016465.1 for* CFTR* gene (Reads = 470; % A = 99.36%). (c) Sequencing electropherograms of patient showing the substitution of T to A at nucleotide 1043 at exon 8 (the arrow indicates the mutant nucleotide).

The emerging NGS allowed us to analyze the full* CFTR* coding region and exon/intron junctions. “*CFTR* scanning” showed a homozygous replacement from T>A at position 1043 in the coding region. Codon 348 (ATG) changed to AAG, causing an amino acid substitution from methionine to lysine (p.M348K) (Figure 1(a)), which is not conservative, since methionine (an apolar amino acid) was replaced by the lysine (a polar amino acid). This result was then confirmed by Sanger sequencing (Figure 1(b)).

## 3. Discussion

To date, over 1900* CFTR* mutations and 300 polymorphisms have been reported (http://www.genet.sickkids.on.ca/app). As widely reported in the literature, the frequencies of* CFTR* mutations are very different depending on the geographical area [5]. The simplest and rapid approach for mutational research involves usage of a defined panel of mutations, able to cover more than 80% of disease risk in the population. However, high* CFTR* genetic heterogeneity is a limiting factor when this approach is used. In fact, mutation panels included in various commercially available kits cannot completely cover all geographical worldwide areas. Therefore, the low detection rate can provide not highly informative first level cystic fibrosis screening assay, above all in absence of family risk situations, such as in case of patients who require CFTR genetic testing before IVD procedure. In these conditions the probability of a CFTR missing test is very high. In fact, since 2003, we have screened more than 9,000 individuals (90% of which was evaluated only in the context of sterility or IVD screening programs): the great part of them (about 96%) resulted as negative at first level* CFTR* testing. Although the large amount of negative CFTR results may be justified by a preventive approach before IVD procedures, we should also take into account that all these individuals were analyzed with commercial kit able to cover a risk ratio close to 85%. Therefore, we cannot exclude the presence of other variants or mutations, not included in the kit employed, within about 8100 individuals screened since 2003. Consequently, in order to provide a complete* CFTR* gene scanning, we analyze the full coding region and exon/intron junctions by Sanger sequencing. Moreover, massive parallel sequencing (MPS) is a novel and efficient strategy that can replace other current low-throughput and time-consuming molecular methods. In fact, MPS approach allows simultaneous complete molecular analysis of different genes in the same run, reducing costs and turnaround time. In this context, our laboratory workflow is able to analyze* BRCA1/2*,* CFTR*, and familiar hypercholesterolemia genes in the same 454 Junior run. For this reason, we decided to introduce NGS as a valid alternative method to* CFTR* gene Sanger sequencing. Further advantage of NGS technology is its flexibility, since it can be coupled also with homemade bioinformatics tools, thus strongly reducing the cost of commercial software: therefore, in our experience, MPS represents reliable and robust approach for the molecular diagnostics of CF and CFTR-RD [6].

Through NGS, we identify the first Italian patient homozygous for the p.M348K* CFTR* mutation. This amino acidic substitution in M6 domain of CFTR protein is nonconservative: methionine is replaced by lysine. This mutation was previously identified: (a) as innocuous polymorphism in a cystic fibrosis at-risk family [7]; (b) in compound heterozygosis with L346P in a Cypriot male individual [8]; and (c) in homozygote boy with respiratory symptoms and failure to thrive [9]. As reported by Hentschel et al. [9], clinical relevance of p.M348K mutation remains unclear. In this regard, the clinical and functional translation of* CFTR* (CFTR2) project represents a novel approach for clinical and functional annotation of mutations identified in disease-causing genes [10]. In fact, CFTR2 will help interpretation of individual genotype-phenotype correlation as for p.M348K, confirming the hypothesis of nonpathogenic role of this variant, although it is still classified as a disease-causing mutation in the Human Genome Mutation Database.

Furthermore, we underline that p.M348 amino acidic residue is completely conserved in 7 species analyzed suggesting evolutionary pressure (data not shown). Since 348 residues are located in the transmembrane M6 domain, which have an important role in the regulation of pore function, the structure of the CFTR protein might be altered, possibly determining a pathological effect due to replacement of the methionine to lysine. Since the transmembrane M6 domain consists of only 3 polar and 16 nonpolar amino acids, amino acid change from methionine to lysine (from polar-to-nonpolar) could not affect chloride channel structure because the other 15 amino acid residues could guarantee the functional structure of the hydrophobic M6 domain: we speculate that this mechanism could explain neutral effect of this mutation on CFTR protein.

In conclusion, based on the clinical features of the patient, we can confirm the data published by D'Apice et al. and Hentschel et al. who considered p.M348K as nonpathogenic variant. In fact our individual did not show any feature of CF or other symptoms related to mild CF form and/or CF-related disorders.

Considering the phenotype and the clinical feature of our proband, we can be able to answer to question addressed by Hentschel et al. in the title of their paper responding that p.M348K is not a disease-causing mutation but a benign rare polymorphism.
